# Exploring the Negative Influence of Commercial Determinants on Oral Health: A Scoping Review

**DOI:** 10.1155/ijod/1825389

**Published:** 2026-08-27

**Authors:** Sanjay Singamsetti, Ramya Shenoy, Violet D’Souza, Harsh Priya

**Affiliations:** ^1^ Department of Public Health Dentistry, Manipal College of Dental Sciences Mangalore, Manipal Academy of Higher Education, Manipal, India, manipal.edu; ^2^ Department of Dental Clinical Sciences, Dalhousie University, Halifax, Canada, dal.ca; ^3^ Division of Public Health Dentistry, Centre for Dental Education and Research, All India Institute of Medical Sciences (AIIMS), New Delhi, India, aiims.edu

**Keywords:** advertising, alcohol industry, commercial determinants, corporate determinants, corporate practices, marketing, oral health, professional corporations, sugar industry, tobacco industry

## Abstract

**Introduction:**

The commercial determinants of oral health refer to the strategies and practices of industries, especially those involving sugar, tobacco, and alcohol, that influence population behaviors and health outcomes. These determinants encompass the provision and promotion of goods and services for profit, including marketing, pricing, product availability, and the broader commercial environment in which these activities occur.

**Objective:**

To understand the extent and types of evidence available on the negative influences of commercial determinants on oral health, map the literature, identify key concepts, and determine research gaps.

**Methods:**

This scoping review was conducted in accordance with the Joanna Briggs Institute (JBI) methodology and reported as per the PRISMA‐ScR guidelines. A comprehensive search was conducted across PubMed, Scopus, Embase, and Web of Science databases using standardized keywords as per the PCC framework. Sanjay Singamsetti synthesized the data narratively and presented them with a PRISMA‐ScR flow diagram.

**Results:**

Twenty articles out of 626 records met the inclusion criteria and examined the influence of beverages and food marketing on children’s oral health behaviors and dietary choices. Studies have shown an association between market exposure and increased consumption of cariogenic products. Content analysis revealed a high prevalence of food advertising featuring unhealthy products on television and in children’s media. Multiple studies have highlighted corporate strategies that influence dietary habits and oral health outcomes across diverse populations, including developed and developing nations.

**Conclusions:**

Collectively, the evidence indicates that commercial actors—primarily those marketing sugary foods and beverages—play a substantial role in shaping dietary behaviors associated with oral disease. Children and adolescents were consistently identified as the primary targets and the most susceptible population. Marketing operates through multiple channels, including broadcast media, product packaging and labeling, children’s magazines, and point‐of‐sale (POS) promotions, contributing to increased sugar intake, increasing caries risk, and widening oral health inequities.

## 1. Introduction

Oral health is a vital component of overall health and well‐being, yet it often receives less attention than other health conditions do. Oral diseases are strongly influenced by modifiable risk factors, including hygiene practices, diet, alcohol use, smoking, and stress [1]. Beyond these behavioral factors, growing evidence highlights the significant role of commercial forces in shaping oral health outcomes. The World Health Organization recognizes that social determinants of health, nonmedical factors shaped by the conditions in which people are born, grow, live, work, and age—have a profound effect on health outcomes [1]. Commercial determinants operate within this broader context, reflecting how commercial activities and market forces influence population health [2].

The commercial determinants of oral health refer to the strategies and practices of industries, especially those involving in sugar, tobacco, and alcohol that influence population behaviors and health outcomes [2]. They operate through several mechanisms: (1) marketing and promotional strategies that shape consumer awareness and preferences; (2) product pricing and availability that influence accessibility; (3) corporate lobbying that affects health policy; and (4) corporate social responsibility (CSR) initiatives that enhance brand image while continuing to promote potentially harmful oral health products [2]. The sugar industry clearly illustrates this influence. Sugar consumption is a major etiological factor for dental caries, and its global availability and desirability are reinforced through systemic marketing, product placement, and pricing strategies [3], exemplifying how commercial determinants impact oral health. Similar patterns are observed in the tobacco and alcohol industries, which continue to affect oral health through products known to be harmful, supported by aggressive marketing practices that often target vulnerable populations, including children [4].

Food advertising is a particularly pervasive commercial determinant [5, 6]. Research consistently shows that television advertising for high‐sugar foods and beverages shapes children’s dietary choices, snacking behaviors, and food preferences [5, 6]. Marketing strategies—ranging from pervasive messages to appealing product design—actively influence consumer perceptions and behaviors, contributing to dietary patterns that increase oral disease risk [5].

Understanding these determinants is essential because they represent modifiable factors that can be addressed through policy intervention. Upstream regulatory measures targeting commercial practices have the potential to reduce oral disease burden and mitigate health inequities. Given that, we aimed to review the literature systematically to gather, identify, and map the extent and nature of existing evidence on negative influences of commercial determinants of oral health. A scoping review is the most appropriate approach as it allows for a broad exploration of the literature on this topic. The lack of scoping reviews on this topic in the literature prompted us to carry out this scoping review. Mapping diverse sources of evidence provides a comprehensive overview of current knowledge while simultaneously enabling the identification of gaps and areas requiring further research.

## 2. Methods

### 2.1. Study Design and Protocol

This scoping review was conducted in accordance with the Joanna Briggs Institute (JBI) methodology and reported as per the PRISMA‐ScR guidelines [7] (Supporting Information 1: File S1). The protocol was registered in the OSF Registry (DOI: 10.17605/OSF.IO/E8Q43).

Review question: the current scoping review was conducted to answer the following review question: “What are the negative influences of commercial determinants on oral health among the general population?”

### 2.2. Search Strategy

A comprehensive search was performed across four major health‐related databases—PubMed, Scopus, Embase, and Web of Science—based on the PCC framework, with initial searches conducted on April 4 and April 30, 2025, and an updated search carried out on May 20, 2026. The key words are population: Studies involving all age groups and populations; concept: negative influence of commercial determinants, marketing, corporate practices, product availability, advertising, lobbying, and impact on oral health outcomes, including the prevalence of oral diseases; and context: global studies, including various settings such as urban and rural areas.

Search strategies were developed iteratively using relevant text words, MeSH terms, and index terms related to commercial determinants and oral health. The search contains the original search plus an updated search to keep it up‐to‐date. The complete search strategy and its results are given in Table 1.

**Table 1:** Search strategy and results.

Database	Date	Search string	Result
PubMed	04/04/2025	((“commercial determinants” [text word] OR (“Professional Corporations”[MeSH terms] OR “corporate determinants” [text word] OR “corporate practices” [text word]) OR (“Tobacco Industry” [MeSH terms] OR “Tobacco Industry” [text word]) OR “sugar industry” [text word] OR “alcohol industry” [text word] OR (”Marketing“ [MeSH terms] OR “Marketing” [text word] OR (“Advertising” [MeSH terms] OR “Advertising”[text word]))) AND (“Oral Health” [MeSH terms] OR “Oral Health” [text word])) AND ((fft[filter]) AND (2014:2025[pdat]))	87
PubMed	20/05/2026	((“commercial determinants” [text word] OR (“Professional Corporations”[MeSH terms] OR “corporate determinants” [text word] OR “corporate practices” [text word]) OR (“Tobacco Industry” [MeSH terms] OR “Tobacco Industry” [text word]) OR “sugar industry” [text word] OR “alcohol industry” [text word] OR (“Marketing” [MeSH terms] OR “Marketing” [text word] OR (“Advertising” [MeSH terms] OR “Advertising” [text word] OR “Lobbying” [MeSH terms]))) AND (“Oral Health” [MeSH terms] OR “Oral Health” [text word])) AND (y_1[filter])	18
Scopus	04/04/2025	(TITLE‐ABS‐KEY (“commercial determinants”) OR (INDEXTERMS (“Professional Corporations”) OR TITLE‐ABS‐KEY (“corporate determinants”) OR TITLE‐ABS‐KEY (“corporate practices”)) OR (INDEXTERMS (“Tobacco Industry”) OR TITLE‐ABS‐KEY (“Tobacco Industry”)) OR TITLE‐ABS‐KEY (“sugar industry”) OR TITLE‐ABS‐KEY (“alcohol industry”) OR (INDEXTERMS (marketing) OR TITLE‐ABS‐KEY (marketing) OR (INDEXTERMS (advertising) OR TITLE‐ABS‐KEY (Advertising)))) AND (INDEXTERMS (“Oral Health”) OR TITLE‐ABS‐KEY (“Oral Health”)) AND PUBYEAR > 2013 AND PUBYEAR < 2026	153
Scopus	20/05/2026	(TITLE‐ABS‐KEY (“commercial determinants”) OR (INDEXTERMS (“Professional Corporations”) OR TITLE‐ABS‐KEY (“corporate determinants”) OR TITLE‐ABS‐KEY (“corporate practices”)) OR (INDEXTERMS (“Tobacco Industry”) OR TITLE‐ABS‐KEY (“Tobacco Industry”)) OR TITLE‐ABS‐KEY (“sugar industry”) OR TITLE‐ABS‐KEY (“alcohol industry”) OR (INDEXTERMS (marketing) OR TITLE‐ABS‐KEY (marketing) OR (INDEXTERMS (advertising) OR TITLE‐ABS‐KEY (advertising) OR TITLE‐ABS‐KEY (lobbying)))) AND ( INDEXTERMS (“Oral Health”) OR TITLE‐ABS‐KEY (“Oral Health”)) AND PUBYEAR > 2024 AND PUBYEAR < 2027	28
Embase	04/04/2025	(‘commercial determinants’:ti,ab,kw OR ‘professional corporations’:ti,ab,kw OR ‘corporate determinants’:ti,ab,kw OR ‘corporate practices’:ti,ab, kw OR ‘tobacco industry’/exp OR ‘tobacco industry’:ti,ab,kw OR ‘sugar industry’/exp OR ‘sugar industry’:ti,ab,kw OR ‘alcohol industry’/exp OR ‘alcohol industry’:ti,ab,kw OR ‘marketing’/exp OR marketing:ti,ab,kw OR ‘advertising’/exp OR advertising:ti,ab,kw) AND (‘dental health’/exp OR ‘oral health’:ti,ab,kw) AND ([embase]/lim OR [medline]/lim) AND [2015–2025]/py	118
Embase	20/05/2026	(‘commercial determinants’:ti,ab,kw OR ‘professional corporations’:ti,ab,kw OR ‘corporate determinants’:ti,ab,kw OR ‘corporate practices’:ti,ab,kw OR ‘tobacco industry’/exp OR ‘tobacco industry’:ti,ab,kw OR ‘sugar industry’/exp OR ‘sugar industry’:ti,ab,kw OR ‘alcohol industry’/exp OR ‘alcohol industry’:ti,ab,kw OR ‘marketing’/exp OR marketing:ti,ab,kw OR ‘advertising’/exp OR advertising:ti,ab,kw OR ‘Lobbying’:ti,ab,kw) AND (‘dental health’/exp OR ‘oral health’:ti,ab,kw) AND ([embase]/lim OR [medline]/lim) AND [2025–2026]/py	34
Web of Science	30/04/2025	((TS = “commercial determinants” OR (TS = “Professional Corporations” OR TS = “corporate determinants” OR TS = “corporate practices”) OR TS = “Tobacco Industry” OR TS = “sugar industry” OR TS = “alcohol industry” OR TS = marketing OR TS = advertising) AND TS = “Oral Health”) AND (PY = = (“2025” OR “2024” OR “2022” OR “2021” OR “2020” OR “2017” OR “2023” OR “2019” OR “2018” OR “2016” OR “2015” OR “2014”) AND LA = = (“ENGLISH”) AND TASCA = = (“DENTISTRY ORAL SURGERY MEDICINE” OR “ECONOMICS” OR “ETHICS” OR “FOOD SCIENCE TECHNOLOGY” OR “HEALTH CARE SCIENCES SERVICES” OR “HEALTH POLICY SERVICES” OR “MULTIDISCIPLINARY SCIENCES”))	139
Web of Science	20/05/2026	((TS = “commercial determinants” OR (TS = “Professional Corporations” OR TS = “corporate determinants” OR TS = “corporate practices”) OR TS = “Tobacco Industry” OR TS = “sugar industry” OR TS = “alcohol industry” OR TS = marketing OR TS = advertising OR TS = lobbying) AND TS = “Oral Health”) query link: https://www.webofscience.com/wos/woscc/summary/75b949de-48df-42a0-8837-b49cec194eaa-01bdc77d9d/relevance/1	34

### 2.3. Search Terms Employed

Commercial determinants, corporate determinants, corporate practices, professional corporations, tobacco industry, sugar industry, alcohol industry, lobbying, marketing, advertising, oral health, and dental health.

### 2.4. Eligibility Criteria

#### 2.4.1. Inclusion Criteria

##### 2.4.1.1. Population

Studies involving all age groups and populations are needed.

##### 2.4.1.2. Concept

Studies addressing the negative influence of commercial determinants on oral health, such as marketing, corporate lobbying, product pricing, or CSR initiatives. Articles that explore oral health outcomes within social and economic contexts are included.

##### 2.4.1.3. Context

Research was conducted globally and regionally.

##### 2.4.1.4. Types of Sources

Quantitative, qualitative, or mixed‐method studies, including systematic reviews and meta‐analyses. Peer‐reviewed journal articles were used. Studies were published between 2014 and May 2026. Research published in English are included.

#### 2.4.2. Exclusion Criteria

##### 2.4.2.1. Population

Studies focusing exclusively on clinical populations (e.g., patients with specific diseases or conditions) without a connection to commercial determinants.

##### 2.4.2.2. Concept

Research in experimental or laboratory settings is disconnected from societal or market impacts. Animal studies. Articles that do not address commercial determinants or focus only on biological/clinical aspects of oral health are excluded. Studies limited to technical advancements in dental technology or treatments without a commercial dimension are excluded. Studies unrelated to oral health or that mention commercial determinants only tangentially are excluded.

##### 2.4.2.3. Types of Sources

Opinion pieces, editorials, or anecdotal evidence without robust methodology are excluded. Publications without empirical or theoretical contributions to the topic are excluded. Studies published outside the selected time range. Non‐English articles are excluded. Reviews that are not systematic reviews and meta‐analyses are excluded.

### 2.5. Study Selection Process

All identified records were imported into Rayyan.ai [8], where duplicates were removed. Two independent reviewers (Sanjay Singamsetti and Ramya Shenoy) screened title and abstracts against the eligibility criteria, followed by a full‐text assessment of potentially relevant articles. Disagreements were resolved through discussion, and no third reviewer was involved. Reasons for exclusion were documented to ensure transparency.

### 2.6. Data Extraction

Data were extracted by two independent reviewers, Sanjay Singamsetti and Ramya Shenoy, using Rayyan.ai [8]. The extracted information included study characteristics (author, year, design, and country), participant demographics (age, gender, and socioeconomic status), type of commercial determinant assessed, context (setting and population), methodological approaches, and key findings regarding commercial determinant impacts on oral health. The extraction tool was piloted on three articles and modified on the basis of the authors’ feedback. All the studies and their extracted data included in this review can be found in Supporting Information 2: File 2.

### 2.7. Data Synthesis and Presentation

The extracted data were synthesized narratively. Findings were mapped to illustrate the scope of available evidence, the populations studied, the commercial actors involved, and the oral health outcomes examined. The results are presented accompanying narrative summaries, tables, and diagrams.

## 3. Results

### 3.1. Study Selection

**Figure 1:**
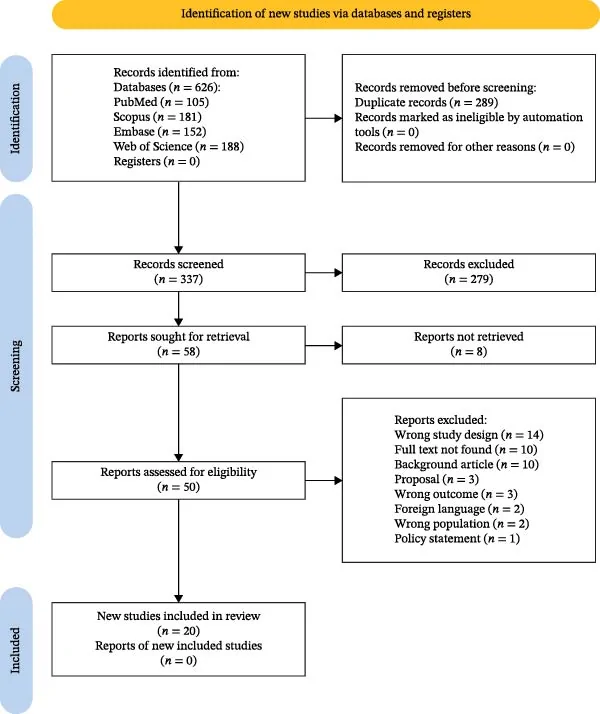
PRISMA flow diagram [9] depicting the process of article selection for the scoping review.

The search identified 626 records across four databases. After 289 duplicates were removed, 337 articles were screened by title and abstract. Of these, 50 full‐text articles underwent full‐text reviews, and 20 articles met the eligibility criteria for inclusion. The most common reasons for exclusion were lack of focus on commercial determinants of oral health, insufficient analysis of commercial influences, non‐English publication, or unavailable full text. The study selection process is summarized in Figure 1 [9].

### 3.2. Characteristics of Included Studies

The 20 included studies (2014–2026) are presented in Table 2. The study designs included are content analysis (*n* = 8), quantitative (*n* = 3), cross‐sectional survey (*n* = 2), qualitative (*n* = 2), randomized controlled trial (*n* = 2), mixed‐methods (*n* = 1), and systematic review (*n* = 2) studies. The studies were conducted across multiple settings, including the United Kingdom, Australia, India, Pakistan, Iran, Bulgaria, Greece, and South Australia, with two studies analyzing evidence across multiple countries (e.g., infant food labeling across 13 countries and a global systematic review/meta‐analysis). Although pricing, corporate lobbying, and CSR were included in the eligibility criteria, no studies addressing these determinants met the inclusion criteria.

**Table 2:** The general characteristics of the included studies.

Author, year	Country	Study design	Population studied	Commercial determinant examined	Key oral health‐related outcome(s)
Chapman et al., 2014 [10]	United Kingdom	Content analysis	Children	Food marketing in children’s magazines, providing free gift	Promotion of high‐sugar foods
Movahhed et al., 2014 [11]	Iran	Content analysis	Children	Television food advertising	Frequency of cariogenic food ads
Somasundaram et al., 2014 [12]	India	Content analysis	Children/adolescents	Television advertisements, magazines/newspaper, posters/banners. Use of favorite models/actors, music, free gifts	Dental caries prevalence
Gatou et al., 2014 [13]	Greece	Quantitative	Children	Television food advertising	Exposure to sugary foods
Gatou et al., 2016 [14]	Greece	Randomized controlled trial	Children	Food advertising exposure	Snack choice and sugar intake
Al‐Mazyad et al., 2017 [3]	United Kingdom	Content analysis	Children/adolescents	Television food and beverage advertising	Exposure to cariogenic food marketing
Bahuguna et al., 2017 [4]	India	Quantitative	Children	Television food advertising	Dietary behaviors and caries risk
Fairchild at al., 2017 [15]	United Kingdom	Cross‐sectional survey	Children	Sports drink marketing via brand logo and sports ambassadors/sponsorship	Exposure to sugary beverages
Arora et al., 2018 [16]	Australia	Content analysis	Children/adolescents	Television food advertising	Sugar‐dense food promotion
Pournaghi Azar et al., 2018 [17]	Global (United Kingdom, India, and Greece)	Systematic review and meta‐analysis	Children	Television food advertising	Promotion of cariogenic foods
Khehra et al., 2018 [18]	United Kingdom	Quantitative	Children	Food packaging and labeling	Dental caries risk
Athavale et al., 2020 [19]	India (urban slums)	Mixed methods	Caregivers and young children	Food marketing and availability	Cariogenic feeding practices early childhood caries (ECC)
Arora et al., 2021 [20]	Australia	Qualitative	Parents/caregivers	Television food advertising	Child feeding decisions early childhood caries (ECC)
Bridge et al., 2021 [21]	Global	Content analysis	Infants/young children	Labeling and marketing strategies of commercial infant foods	Dental caries risk associated with inappropriate feeding and high sugar intake
Doitchinova et al., 2021 [22]	Bulgaria	Cross‐sectional survey	Children/adolescents	Television food advertising	Prolonged TV screen time correlated with increased intensity of dental caries
Kearney et al., 2021 [23]	United Kingdom	Randomized controlled trial	Children	Food advertising exposure	Increased sugar consumption
Poirier et al., 2021 [24]	Australia	Qualitative	Indigenous caregivers	Food availability and misleading marketing	Caries prevalence
Boyland et al., 2022 [5]	Global	Systematic review and meta‐analysis	Children/adolescents	Food marketing	Dietary behaviors and caries risk
Farzeen et al., 2026 [25]	Pakistan	Content analysis	General population	Nicotine pouch POS marketing	Potential oral health implications
Mohammadzadeh et al., 2025 [26]	Iran	Content analysis	General TV audience (children, teenagers, adults)	Television food and beverage advertisements	Prevalence of cariogenic food ads (65.5%); lack of oral hygiene product advertising (0.5%)

### 3.3. Commercial Determinants

Most studies have examined food and nonalcoholic beverage marketing directed at children, including television advertising [3, 4, 11–13, 16, 17, 20, 22, 26], packaging and labeling [18, 21], children’s magazines [10], infant foods, breakfast cereals, and sports drinks [15, 19, 21], and the behavioral effects of food advertising exposure [4, 5, 14, 23]. In total, 19 of the 20 included studies addressed food and beverage marketing. One study focused on tobacco/nicotine marketing at the point‐of‐sale (POS) [25]. None of the included studies investigated alcohol marketing.

### 3.4. Populations Studied

The populations were predominantly infants and children, including indigenous individuals and adolescents (*n* = 14), and one study involving caregivers in South Australia was included [24]. None of the included studies sampled a general adult population without focusing on children.

#### 3.4.1. Food and Beverage Marketing Across Media

Television content analyses consistently revealed a high frequency of advertisements for foods and drinks detrimental to oral health during children’s viewing times and peak hours, often using persuasive techniques such as gifts, cartoon characters, music, and emotional appeals [3, 11, 13, 16, 26]. Beyond broadcast media, covert promotion in children’s magazines and misleading front‐of‐pack (FOP) labeling and packaging—particularly for children’s cereals and commercial infant foods—were shown to normalize sugar‐dense diets (e.g., “no added sugar” claims masking high total sugar content; oversized portion‐size imagery) [10, 18, 21].

#### 3.4.2. Behavioral Effects of Advertising Exposure

Experimental and behavioral studies have shown that exposure to high‐sugar food advertising increased children’s immediate sugar and energy intake and shifted their preferences toward unhealthy snacks, with children who already had dental caries demonstrating greater susceptibility to these effects [14, 23]. Cross‐sectional analyses further reinforced this pattern, showing associations between screen time, exposure to sugary food advertising, and increased caries activity [12, 22].

#### 3.4.3. Socioeconomic and Vulnerable Groups

Studies conducted in socioeconomically disadvantaged settings (e.g., urban slums and low‐income families) and those involving caregivers highlighted heightened vulnerability due to concentrated marketing exposure, the affordability of unhealthy foods, limited access to healthier alternatives, misleading labeling, and structural barriers including racism and restricted access to dental services [19, 20, 24]. Urban–rural comparisons also suggest that children in urban contexts may be more responsive to marketing because of greater product availability and a stronger commercial influence [4].

#### 3.4.4. POS and Emerging Products

A single tobacco/nicotine study revealed widespread availability, low pricing, and youth‐appealing POS promotion of nicotine pouches, signaling an emerging commercial determinant with potential oral health implications [25].

### 3.5. Oral Health Outcomes

Across studies, consistent oral health outcomes included increased preference for and consumption of cariogenic foods and drinks following advertising exposure, higher prevalence of dental caries among exposed children, and stronger advertising effects among children with existing caries or from socioeconomically disadvantaged backgrounds [19, 22, 23].

## 4. Discussion

This scoping review mapped the existing literature on commercial determinants of oral health, identifying 20 relevant studies documenting the ways in which commercial determinants influence oral health outcomes.

### 4.1. Summary and Interpretation

The results of this review indicate that commercial actors influence oral health, particularly through beverage and food marketing targeting children and adolescents. Television content analyses consistently documented a high prevalence of cariogenic product advertising during child‐viewing times, with minimal oral health messaging [3, 11, 13, 16, 22, 26]. Behavioral studies confirmed associations between marketing exposure and increased consumption of harmful dietary products, representing direct pathways to oral disease development [4, 5, 14, 22, 23].

The review identified marketing as the predominant commercial determinant mechanism studied, while other determinants such as pricing, product availability, packaging, and POS promotion were far less frequently examined. Content analyses revealed sophisticated marketing strategies deliberately designed to appeal to children, employing cartoon characters, toys, celebrity endorsements, and appealing packaging—mechanisms recognized in the commercial determinants literature as highly effective in shaping consumer behavior among vulnerable populations [3, 5, 18, 19, 21, 23, 25].

### 4.2. Commercial Determinant Mechanisms

The relationship appears to operate even when consumers possess nutritional knowledge, suggesting that commercial determinants may function through psychological and behavioral mechanisms beyond simple information provision. The evidence indicates multiple mechanisms through which commercial activities may affect oral health, indicating (a) consumer behavior modification through marketing exposure [4, 5, 14, 23]; (b) influence on food and beverage consumption patterns through product promotion and placement [3, 10, 13, 16]; (c) parental and caregiver decision‐making regarding child nutrition [18–21, 24]; (d) health inequities through differential targeting and accessibility across socioeconomic groups [4, 19, 20, 24, 25].

Food marketing represents the most extensively documented commercial determinant [5, 17]. Consistent findings across multiple countries and populations that television advertising for high‐sugar foods is significantly associated with increased consumption patterns provide robust evidence of marketing effectiveness [4, 5, 14, 22, 23]. This relationship operates even when consumers possess nutritional knowledge, suggesting that commercial determinants function through psychological and behavioral mechanisms beyond simple information provision [15, 19, 20].

### 4.3. Vulnerable Populations and Health Equity

Multiple studies have reported that children, adolescents, and indigenous and lower‐income populations, and people represent particularly vulnerable groups for determining commercial impacts. Evidence indicates that children show greater behavioral susceptibility to advertising [4, 5, 14, 22–24]; lower‐income families face greater constraints in accessing health‐promoting alternatives to commercially promoted products [19, 20]; and targeting strategies employed by commercial actors appear to focus on vulnerable populations [3, 10, 13, 16]. These findings support enhanced policy protection for susceptible groups.

### 4.4. Emerging Commercial Determinants

This review identified emerging commercial determinants that require research attention. Novel nicotine products, including nicotine pouches, represent evolving commercial strategies with unknown oral health impacts but demonstrate aggressive marketing [25]. Digital marketing through social media and online platforms remains understudied within the oral health literature despite widespread adoption in youth‐targeted commercial campaigns. Despite the extensive commercialization of oral hygiene and cosmetic dental products, no study has empirically examined their marketing or commercial influence on oral health outcomes. These emerging mechanisms represent gaps in current evidence that require urgent investigation [5, 10, 20, 25].

### 4.5. Strength and Quality of Evidence

The evidence base predominantly comprises descriptive observational studies and content analyses, providing detailed descriptive epidemiological insights into the prevalence of commercial determinants but limited causal evidence. Cross‐sectional associations between marketing exposure and dietary behaviors suggest causal relationships that are plausible from commercial determinants theory but require prospective investigations and experimental designs for confirmation [3, 5, 10, 13, 16, 22, 26]. Few longitudinal studies have examined oral health outcomes over time, and intervention studies evaluating policy impacts are largely absent; together, these gaps limit the ability to establish definitive causal relationships and assess the effectiveness of specific policy interventions [5, 17, 22, 25].

The geographic concentration of research in high‐income countries (for example, the United Kingdom, Australia, and Iran), with limited representation from low‐ and middle‐income settings, restricts understanding of commercial determinant contexts in developing nations where regulatory frameworks may be weaker and commercial actor influence potentially greater. Despite their inclusion in the review, the limited volume of empirical work from these settings highlights a major research gap [5, 19, 25, 26].

### 4.6. Policy Implications

The evidence base supports the consideration of policy responses addressing commercial determinants of oral health. Although the included studies were largely descriptive and provided limited direct policy‐evaluation evidence, they suggest that self‐regulation by commercial actors may be insufficient and that stronger regulatory frameworks may be warranted to protect public health [3, 5, 10, 13, 16, 17]. In line with the broader public health and oral health literature, plausible policy directions include advertising restrictions during child‐viewing times, mandatory health warnings on product packaging, taxes on sugar‐sweetened beverages, restrictions on marketing claims, front‐of‐package labeling, and tighter controls on marketing channels targeting children [3, 5, 10, 13, 15, 18, 21, 25, 27].

The finding that commercial determinants disproportionately impact lower‐income populations suggests that the policy focus should address health equity. Marketing restrictions, pricing policies, and product availability modifications can serve as upstream interventions to reduce the oral disease burden in vulnerable populations [4, 5, 19, 20, 24, 27].

### 4.7. Research Recommendations

Future research should address the following gaps: (1) prospective longitudinal studies examining commercial determinant exposure effects on oral health outcome development; (2) intervention trials evaluating the effectiveness of policy modifications in reducing commercial determinant impacts; (3) economic analyses assessing the impact of commercial determinants on healthcare costs and the cost‐effectiveness of policy interventions; (4) qualitative research examining commercial determinant mechanisms and consumer decision‐making processes in diverse cultural contexts; (5) digital marketing research examining the effects of social media and online platforms on oral health behaviors; (6) implementation science research examining the feasibility and effectiveness of policy interventions in real‐world settings; (7) comparative effectiveness research examining the relative impacts of different regulatory approaches on oral health outcomes; (8) research employing mixed‐methods approaches that integrate quantitative outcome data with a qualitative understanding of commercial determinant mechanisms and consumer experiences to enhance the quality of evidence for policy development; (9) research addressing the influence of commercial determinants (such as lobbying, CSR, and pricing) that are employed by tobacco, alcohol, oral and dental hygiene Industries on oral health; (10) future research should evaluate how health‑oriented taxation of sugar‑sweetened beverages can be designed, implemented, and monitored to both reduce consumption and sustainably finance oral health integration within national noncommunicable disease (NCD) and universal health coverage (UHC) frameworks, including assessments of cost‑effectiveness and distributional equity.

### 4.8. Limitations of the Study

A key limitation of this study is its reliance on only four primary health‐related databases. This may have led to the exclusion of some relevant studies focused on commercialization and the corporate sector. In addition, non‐English and gray literature was not considered because of constraints related to time, resources, and translation, potentially limiting the inclusion of region‐specific research. Critical appraisal was not carried out in this study as it is not necessary according to JBI guidelines. While the focus on the peer‐reviewed sources helps ensure methodological rigor and data reliability, future reviews should consider including multilingual and gray literature to achieve a more comprehensive synthesis.

## 5. Conclusions

Collectively, the evidence indicates that commercial actors—primarily those marketing sugary foods and beverages—play a substantial role in shaping dietary behaviors associated with oral disease. Children and adolescents were consistently identified as the primary targets and the most susceptible population. Marketing operates through multiple channels, including broadcast media, product packaging and labeling, children’s magazines, and POS promotions, contributing to increased sugar intake, increasing caries risk, and widening oral health inequities. None of the included studies examined the impact of commercial determinants on adult oral health outcomes or alcohol marketing or assessed long‐term oral health outcomes beyond adolescence.

## Author Contributions

**Sanjay Singamsetti**: conceptualization, data curation, formal analysis, methodology, writing the original draft, reviewing, editing. **Ramya Shenoy**: conceptualization, data curation, formal analysis, methodology, reviewing, editing. **Violet D’Souza**: formal analysis, writing of original draft, reviewing, editing. **Harsh Priya**: reviewing, editing, supervision.

## Funding

Open access funding was provided by the Manipal Academy of Higher Education, Manipal.

## Disclosure

All authors have read and approved the final version of the manuscript. Ramya Shenoy had full access to all the data in this study and takes complete responsibility for the integrity of the data and the accuracy of the data analysis. All AI‐generated content was reviewed, edited, and verified by the authors, who take full responsibility for the accuracy and originality of the manuscript.

## Ethics Statement

As this scoping review synthesized evidence from previously published and publicly available sources, ethical approval was not required.

## Conflicts of Interest

The authors declare no conflicts of interest.

## Supporting Information

Additional supporting information can be found online in the Supporting Information section.

## Supporting information


**Supporting Information 1** file 1: PRISMA‐ScR checklist.

**Supporting Information 2** file 2: Data extraction table.

## Data Availability

The authors confirm that the data supporting the findings of this study are available within its Supporting Information.
